# Mr. Griffiths, on Inoculation

**Published:** 1805-07-01

**Authors:** J. Griffiths

**Affiliations:** No. 75, Grosvenor Street


### *To the Editors of the Medical and Physical Journal.*

GENTLEMEN,

FROM a thorough conviction of the utility and importance of the vaccinating system, I have been one of its earliest and most strenuous advocates. I have myself inoculated upwards of five thousand, and have supplied the virus to other practitioners to a great extent.

Yet, as truth is my only object, I take the earliest opportunity of informing you, that two of my patients, who went through the disease in the most satisfactory manner, one nearly five years ago, and the other about a year and a half since, have recently undergone a decided natural small-pox.

How far two instances out of five thousand, are to be considered as an argument against the cow-pock, it is not my intention to discuss here; I simply state the fact for the consideration of my professional brethren.

I am, &c.

*No.* 75, *Grosvenor Street, June* 21, 1805. J. GRIFFITHS.

