# Erratum

**Published:** 1805-08-01

**Authors:** 


### ERRATUM.

No. 77, p. 6, last line but one, instead of *this is*, read, *this, if local, is, &c.*

